# Abdominal Tuberculosis in a Child Presenting with Radiological Evidence of Pneumatosis Intestinalis and Portal Venous Gas

**DOI:** 10.3329/jhpn.v28i6.6612

**Published:** 2010-12

**Authors:** Handan Alp, Zerrin Orbak, Oguzhan Sepetcigil, Mecit Kantarci, Ibrahim Kartal

**Affiliations:** ^1^ Department of Pediatrics; ^2^ Department of Pediatric Endocrinology and Metabolism; ^3^ Department of Radiology, Faculty of Medicine, Ataturk University, Erzurum, Turkey

**Keywords:** Case studies, Child, Pneumatosis intestinalis, Tuberculosis, Abdominal, Turkey

## Abstract

Pneumatosis intestinalis, in association with portal venous gas, is a rare finding in children and young adults. In radiological studies, it is characterized by gas-filled cysts within the bowel-wall. It is often a sign of the serious significant underlying illness and is associated with a poor prognosis. A case of pneumatosis intestinalis and portal venous gas associated with abdominal tuberculosis in a child is presented here. Despite responding well to anti-tubercular treatment, he died suddenly at home, two months after discharge. It is recommended that cases with pneumatosis intestinalis should be carefully observed, although symptoms appear to be improving.

## INTRODUCTION

Pneumatosis intestinalis (PI) or pneumatosis cystoides intestinalis, in association with portal venous gas, is a rare finding in children and young adults. It is characterized by gas-filled cysts within the bowel wall (1, 2).

Du Vemoi first described PI in 1783 (3). It is not a disease in itself but a radiological sign of an underlying problem. Many different causes of PI have been anticipated, including mechanical and bacterial causes. Approximately 85% of causes are thought to be secondary to co-existing disorders of the gastrointestinal tract or the respiratory system (4, 5).

The relative importance of these conditions to PI in non-neonates is not clear because of the most previous reports which share an underlying diagnosis (6). In neonates, PI is commonly caused by necrotizing enterocolitis (4).

We present here the first case of PI and portal venous gas associated with abdominal tuberculosis in a Turkish child and a brief review of the literature.

## CASE REPORT

An eight-year-old child was admitted to our hospital (Medical Faculty Hospital, Ataturk University) in December 2007, with a two-year history of increasing abdominal distention, abdominal pain, weight loss, anorexia, and malaise. He had been seen and investigated by several paediatricians in other hospitals, including a diagnostic laparotomy. Histopathological diagnosis from the laparotomy was reported as ‘reactive lymph nodes’. He had no significant past medical history but there was a strong family history of tuberculosis: his grandmother, aunt, and maternal uncle had pulmonary tuberculosis, and his maternal uncle had died from pulmonary tuberculosis one year ago.

On examination, he looked ill. He was apyrexial with no lymphadenopathy and had no Bacillus Calmette-Guerin scar. His height was on 50th percentile but his weight was only on 3rd percentile. Abdominal examination revealed diffuse distention and tenderness. The rest of the examination was unremarkable.

On investigation, skin test for tuberculosis with 5 TU was negative. Haemoglobin and WBC were normal. Erythrocyte sedimentation rate was 20 mm/h. Serum adenosine deaminase level was high (34.80 U/L) (normal range: 5.00–20.00 U/L). Antigliadin IgA, IgG, and anti-endomysial antibodies were negative. Serum IgG, IgA, and IgM levels were normal. P-ANCA, ASCA, ANA, and anti-ds-DNA were negative. He was HIV-negative.

**Fig. 1.**
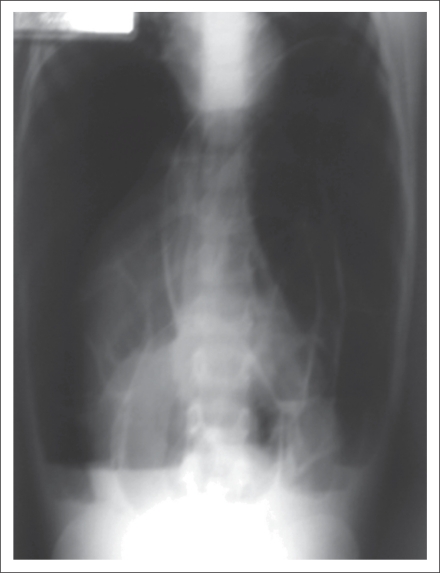
Plain abdominal roentgenogram showing dilated loops of bowel with extensive intramural gas

Plain abdominal x-rays showed dilated loops of bowel with extensive intramural gas, and free intra-abdominal gas was present (Fig. 1). An abdominal ultasound demonstrated the presence of dilated intestinal loops and extensive echogenic material within the portal system of the liver in keeping with portal venous gas.

**Fig. 2.**
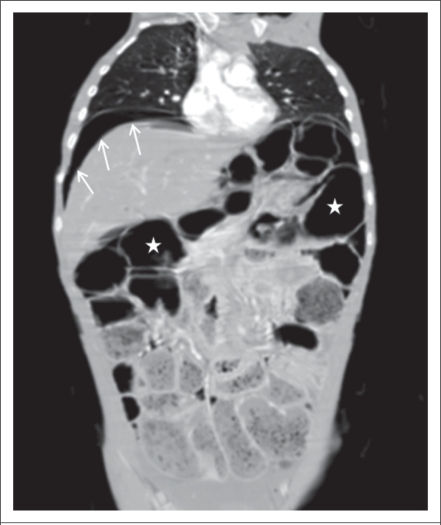
Coronal CT image showing extensive small-bowel pneumatosis (stars), pneumoperitoneum (white arrows), and axial CT image

**Fig. 3.**
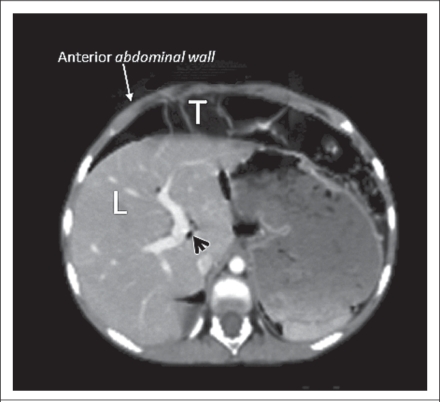
Coronal CT image showing hepatic portal venous gas (black arrow-head) and chilaiditi syndrome

An abdominal coronal and axial compound tomography (CT) images confirmed extensive small-bowel pneumatosis, pneumoperitoneum, hepatic portal venous gas, and chilaiditi syndrome (Fig. 2 and 3).

CT=Computed tomography

CT=Computed tomography;
L=Liver;
T=Transfer colon

We performed upper and lower gastrointestinal endoscopy. In the upper gastrointestinal tract, antral gastritis and duedonitis were detected. In the lower gastrointestinal endoscopy, we could not pass sigmoid. So, no samples were sent for biopsy.

Stool samples and gastric aspirates were negative for acid-fast bacilli. Polymerase chain reaction (PCR) for *Mycobacterium tuberculosis* was positive in the histopathologic specimens from the mesenteric lymphnodes. Study of chest x-ray did not show any evidence of active or healed lesions of tuberculosis.

The patient appeared relatively stable with no clinical evidence of abdominal sepsis or bowel infarction. A surgical opinion recommended conservative treatment. His feeding was insufficient at admission but his appetite increased with treatment.

**Fig. 4.**
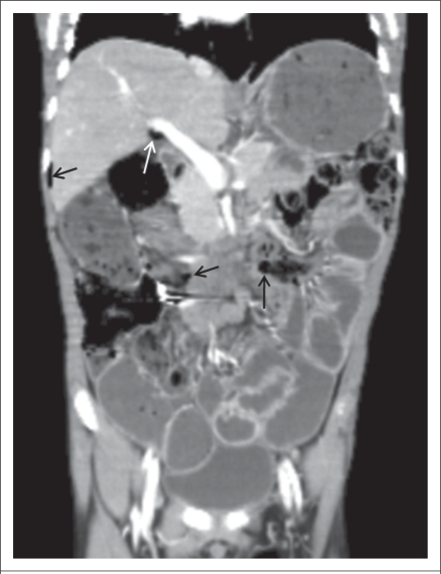
Coronal abdominal CT image showing a little small-bowel pneumatosis, pneuperitoneum (black arrows), and hepatic portal venous gas (white arrow)

**Fig. 5.**
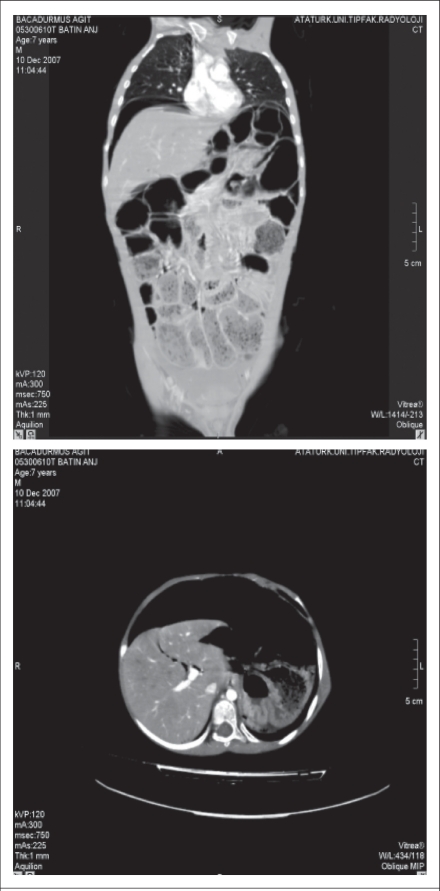
Abdominal CT before treatment

**Fig. 6.**
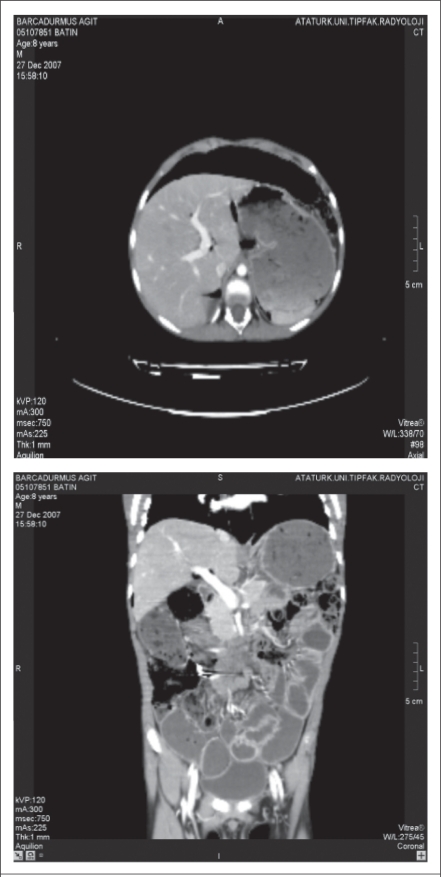
Abdominal CT after treatment

A diagnosis of abdominal tuberculosis was made based on the positive PCR in the histopathologic specimen and positive family history of tuberculosis, and a four-drug anti-tuberculosis regimen (streptomycin+isoniazid+rifampicin+pyrazinamide) was started. Over the first three weeks, clinical symptoms resolved, abdominal distention decreased, and he gained 2 kg in weight. After three weeks, abdominal CT showed a little improvement in the small-bowel pneumatosis, pneuperitoneum, and hepatic portal venous gas (Fig. 4 and 5). Because of his response to therapy (Fig. 6), he was discharged. However, two months after discharge from our hospital, we learned that he died suddenly at home. The cause of death was not known, and an autopsy was not performed.

CT=Computed tomography

CT=Computed tomography

Computed tomography

## DISCUSSION

PI is a radiological sign characterized by cystic or linear collections of gas in the subseroza or submucosa of the gastrointestinal tract (3, 7). Three possibilities have been proposed as the source of the gas within the wall of the gastrointestinal tract: (a) Intraluminal GI gas, (b) bacterial production of gas, and (c) pulmonary gas (3, 5). When neglected, the intramural gas eventually drains into the mesenteric veins and subsequently into the portal venous system. Hepatic portal venous gas (HPVG) usually indicates an ominous prognosis, and it has a mortality rate of 75–85% (8). The pathogenesis of HPVG is not fully understood. Two theories—mechanical and bacterial—have been proposed. The mechanical theory proposes that gas dissects into the bowel-wall from either the intestinal lumen or the lung. Also, mucosal damage allows intraluminal gas to enter the venous system (9, 10). The bacterial theory proposes that gas-forming bacilli enter the submucosa through mocosal rents and produce gas within the intestinal wall and then enter the portal vein (11, 12).

Little information is available for aetiology, diagnosis, and management of PI in older children, although the condition has been well-described in adults: of 919 cases reported in the literature, only 74 (8%) patients were aged less than 20 years (11, 13). *M. tuberculosis* is a rare cause of the infectious conditions underlying PI (5). According to our knowledge, there is only one reported case of PI associated with tuberculosis in the world (14).

We report the first case of PI and HPVG with abdominal tuberculosis in a child. Tuberculosis is still a considerable problem in developing countries (15, 16). The spectrum of the disease in children is different from adults, in whom adhesive peritoneal and lymph-nodal involvement is more common (16). Abdominal tuberculosis is a rare presentation of the disease in childhood. It is an important but probably an underestimated clinical problem (16). Diagnosis of abdominal tuberculosis is difficult because it mimics many other abdominal conditions and has protean manifestations, such as abdominal mass, pain, lymphadenopathy, fever, ascites, and weight loss. Since radiological studies and laboratory tests may be non-diagnostic (17–19), as illustrated by our patient, it is important that tuberculosis is always considered in the differantial diagnosis of unusual gastrointestinal presentations. In our patient, the clinical improvement after three weeks of treatment of tuberculosis supported the diagnosis. Unfortunately, he died suddenly at home, and the cause of death was not known. It is possible that the cause of death was gas embolism in our case because there was gas in the portal system.

### Conclusion

We present this case to raise awareness of the diagnosis of abdominal tuberculosis in patients with PI and portal venous gas in endemic regions for tuberculosis or developing countries. PI and portal venous gas are an uncommon condition and often have a poor prognosis. Abdominal tuberculosis may be an unusual presentation in children with pneumatosis and portal venous gas. We recommend that cases with PI should be carefully observed, although symptoms appear to be improving.
